# The Roles of SHCBP1 in Cancer Hallmarks: Molecular Mechanisms and Therapeutic Implications

**DOI:** 10.3390/ijms26188778

**Published:** 2025-09-09

**Authors:** Hye-Youn Kim, Ye-Jin Park, Soyeon Ryu, Suntaek Hong

**Affiliations:** 1 Department of Biochemistry, Lee Gil Ya Cancer and Diabetes Institute, Gachon University College of Medicine, Incheon 21999, Republic of Korea; 2 Department of Health Sciences and Technology, Gachon Advanced Institute for Health Sciences and Technology, Gachon University, Incheon 21999, Republic of Korea

**Keywords:** SHCBP1, cancer hallmarks, therapeutic target, prognostic biomarker

## Abstract

The SHCBP1 (SHC SH2-domain-binding protein 1) is identified as an important regulator of cancer biology, participating in the modulation of multiple cancer hallmarks. Initially discovered as a component of the mitotic midbody essential for cytokinesis, SHCBP1 is now recognized for orchestrating a broad spectrum of oncogenic processes such as persistent proliferation, apoptosis resistance, epithelial–mesenchymal transition, and immune system evasion. This review comprehensively explores the molecular features of SHCBP1, its regulatory networks, and its multifaceted roles in cancer progression. SHCBP1 is commonly overexpressed in diverse cancers, with elevated expression levels strongly associated with more aggressive tumors and unfavorable patient prognosis. Mechanistically, SHCBP1 serves as a potential mediator of oncogenic signaling pathways, thereby regulating mitotic processes, transcriptional alterations, and cytoskeletal reorganization. In addition to its biological functions, SHCBP1 offers translational promise as a prognostic marker and a prospective therapeutic target. Preclinical models indicate that genetic depletion or pharmacologic disruption of SHCBP1 limits tumor growth, increases sensitivity to chemotherapy, and reduces metastatic capacity. Despite significant progress, the development of selective SHCBP1 inhibitors remain challenging areas. This review summarizes SHCBP1’s diverse roles in tumor pathogenesis and outlines future research directions to develop SHCBP1-targeted strategies.

## 1. Introduction

Cancer constitutes a complex disease characterized by the stepwise acquisition of malignant properties that support unregulated cell growth, metastatic capacity, and resistance to anticancer therapies [1,2]. These defining features, collectively termed the “hallmarks of cancer,” encompass sustained proliferative signaling, suppression of growth-inhibitory signals, avoidance of programmed cell death, limitless replicative potential, angiogenesis induction, acquisition of invasive and metastatic traits, perturbation of cellular metabolism, and mechanisms to circumvent immune surveillance [3,4]. In the past twenty years, this framework has profoundly influenced cancer research, facilitating the identification of pivotal molecular regulators that simultaneously impact various aspects of tumor development. These essential effectors attract considerable attention, as they provide promising therapeutic avenues for addressing pathway redundancy and tumor heterogeneity.

Among various emerging regulatory proteins, SHC SH2-domain binding protein 1 (SHCBP1) has garnered increasing attention due to its diverse functions in cancer biology. Initially identified as a murine protein found in activated lymphocytes (mPAL) using yeast two-hybrid screening of T-cell and embryo libraries, SHCBP1 was recognized as a binding partner of the SHC1 SH2 domain that does not require phosphorylation [5]. Further research has shown that SHCBP1 expression is tightly linked to cellular proliferation and becomes upregulated following growth factor stimulation [6,7,8,9]. Under physiological conditions, SHCBP1 is involved in a variety of biological processes such as cytokinesis, neuronal development, spermatogenesis, and growth factor-mediated signaling. Notably, despite its marked homology with Drosophila Nessun Doma, a regulator of T-cell development, mouse genetic knockout experiments have indicated that SHCBP1 is not indispensable for thymocyte development but primarily contributes to effector CD4+ T cell-related inflammatory pathways [10,11]. In addition, SHCBP1 is shown to modulate measles virus replication by binding to the viral C protein or phosphoprotein and influencing viral RNA polymerase function [12].

SHCBP1’s oncogenic properties are confirmed through clinical and laboratory studies. Increased SHCBP1 expression is detected in a wide range of tumors, including breast cancer, non-small-cell lung carcinoma (NSCLC), gastric adenocarcinoma, hepatocellular carcinoma (HCC), leukemias, gliomas, and cutaneous cancers [13,14,15,16,17,18,19]. Notably, high SHCBP1 levels often coincide with more aggressive tumor characteristics and poorer prognosis. Mechanistically, SHCBP1 is involved in multiple facets of tumorigenesis. SHCBP1 drives cell cycle advancement by supporting transitions at both the G1/S and G2/M checkpoints, primarily via regulation of cyclin-dependent kinase (CDK) activity and the dynamics of mitotic spindles [20]. In addition, SHCBP1 fosters metastatic capabilities by promoting epithelial–mesenchymal transition (EMT) and enhancing motility through the activation of several signaling routes [21,22,23]. SHCBP1 also confers resistance to apoptosis by regulating caspase function and upregulating anti-apoptotic molecules [14,15]. Preliminary studies also point to potential contributions to tumor immune escape mechanisms and metabolic adaptation, but these areas remain to be fully clarified [24].

Despite recent significant advancements, there remains a lack of a comprehensive overview that delineates SHCBP1’s multifaceted roles in relation to the hallmark features of cancer. This review is designed to systematically analyze the molecular mechanisms through which SHCBP1 modulates each of the principal cancer hallmarks. We first describe the structural features and regulatory mechanisms that modulate SHCBP1 expression and function, followed by an extensive evaluation of its roles in tumorigenesis. In addition, we provide a critical assessment of SHCBP1’s prognostic biomarker value and review contemporary therapeutic strategies targeting SHCBP1-related pathways. Through the integration of these elements within the established cancer hallmarks framework, this review aims to offer new perspectives on SHCBP1’s function in tumorigenesis and to underscore its therapeutic potential.

## 2. Molecular Characteristics and Regulation of SHCBP1

### 2.1. Genomic Organization and Protein Structure

**Figure 1:**
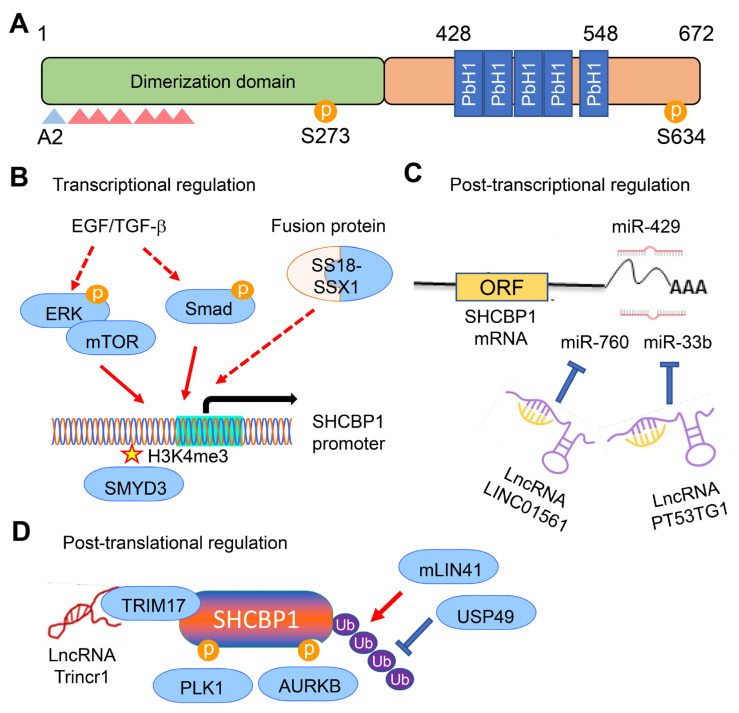
Molecular architecture and regulatory mechanisms of SHCBP1. (**A**) Diagrammatic illustration of SHCBP1 protein organization, depicting key structural domains and sites of post-translational modification. Brown Ⓟ symbols denote experimentally validated phosphorylated serine residues. Blue and red triangles mark predicted N-acetylation sites on alanine and phosphorylation sites on serine residues (5, 7, 31, 42, 44, 47), respectively. The C-terminal region comprises five parallel beta-helix (PbH1) repeat motifs. (**B**) Transcriptional regulation of SHCBP1. Stimulation by EGF-activated ERK or mTOR pathways, TGF-β-induced Smad, and SS18-SSX1 fusion proteins promote SHCBP1 gene expression at the transcriptional level. The histone methyltransferase SMYD3 increases SHCBP1 transcription by catalyzing H3K4me3 epigenetic modification of histones. Dashed arrows indicate indirect regulation of SHCBP1 transcription. (**C**) Post-transcriptional regulation of SHCBP1. MiR-429 downregulates SHCBP1 expression by binding to its 3′-UTR region, leading to mRNA degradation at the post-transcriptional stage. Long noncoding RNAs LINC01561 and PT53TG1 regulate SHCBP1 mRNA stability via sponging activities toward miR-760 and miR-33b, respectively. (**D**) Post-translational modification of SHCBP1. PLK1 and AURKB phosphorylate Ser273 and 634, facilitating nuclear localization and association with growth-promoting proteins, respectively. The stability of SHCBP1 protein is further modulated by mLin41-mediated ubiquitination and USP49-induced deubiquitination at the post-translational level. LncRNA Trincr1 also diminishes SHCBP1 protein stability by interacting with TRIM71 in embryonic stem cells.

The SHCBP1 gene resides on chromosome 16q11.2, comprising 13 exons that encodes a 5.2 kb mRNA translating into 672 amino acids [20]. Structural investigations have demonstrated that SHCBP1 harbors multiple functional domains responsible for various cellular roles, in addition to predicted post-translational modification sites [25,26]. The N-terminal region is characterized by a coiled-coil domain, which is essential for homodimerization and interaction with mitotic apparatus components (Figure 1A). The C-terminal domain includes five parallel beta-helix (PbH1) repeat motifs and a serine phosphorylation site, indicating a role in intracellular signaling and cytoskeleton modulation [9]. While SHCBP1 does not possess innate enzymatic functions, it operates as a fundamental scaffold protein that orchestrates diverse mitogenic and cell cycle pathways, ultimately impacting key cellular events such as proliferation, survival, and developmental processes [27].

### 2.2. Dynamic Subcellular Localization and Cell Cycle Regulation

SHCBP1 displays dynamic changes in its subcellular localization throughout the cell cycle. During interphase, the protein is primarily found in the cytoplasm; however, it relocates markedly to the midbody as cells progress through mitosis. This cell cycle-dependent distribution is stringently controlled by post-translational modifications, with phosphorylation events mediated by polo-like kinase 1 (PLK1) playing a critical role [6,9,28]. Targeted depletion of SHCBP1 using RNA interference in HeLa or A549 cells disrupts the WEE1-pCDC2 regulatory axis, leading to unscheduled mitotic entry and failure of cytokinesis. In clinical samples of HCC, elevated SHCBP1 expression correlates with a heightened mitotic index and increased chromosomal instability, supporting the notion that SHCBP1 overexpression may drive tumorigenesis through impaired mitotic control [16,29].

SHCBP1’s subcellular localization is also influenced by growth factor signaling pathways. Upon epidermal growth factor (EGF) stimulation, SHCBP1 undergoes phosphorylation at Ser273, which prompts its translocation to the nucleus, where it takes part in activating downstream signaling pathways [21,23]. This nuclear translocation in response to growth factor stimulation has been extensively characterized in oncogenic signaling in gastric and bladder cancers, where it plays a role in promoting cell proliferation and invasiveness.

### 2.3. Transcriptional and Post-Transcriptional Regulation of SHCBP1

Interestingly, previous study has shown that SHCBP1 exhibits cell type-specific expression. It is highly expressed in proliferative cells such as activated lymphocytes and stem cells, but almost undetectable in differentiated cells like cardiomyocytes [5]. This distribution pattern indicates that SHCBP1 transcription is tightly regulated by growth factors such as EGF or transforming growth factor β (TGF-β) (Figure 1B) [21,22]. Several studies have recently elucidated transcriptional regulators that modulate SHCBP1 gene activity. Chromatin immunoprecipitation assays performed in breast cancer cells have shown direct association of the histone methyltransferase SMYD3 with the SHCBP1 promoter, facilitating transcriptional induction through H3K4me3 modification [30]. In further support of this regulatory mechanism, a pan-cancer analysis revealed that elevated SHCBP1 expression is significantly associated with increased copy number alterations and reduced DNA methylation of the SHCBP1 gene [31]. For synovial sarcoma, the specific SS18-SSX1 fusion oncoprotein has been shown to contribute to increased SHCBP1 expression, yet the molecular details are not completely defined [32]. This aligns with the well-established capacity of fusion oncoproteins to induce widespread changes in gene transcriptional programs, facilitating tumorigenesis in many cancers [33,34,35].

Recent findings have illuminated that SHCBP1 expression is extensively regulated post-transcriptionally by multiple noncoding RNAs (Figure 1C). In glioma, miR-429 has been validated as a post-transcriptional regulator by binding to the 3′ untranslated region of SHCBP1 mRNA, resulting in translational repression or mRNA decay [36]. Likewise, in NSCLC, the long noncoding RNA (lncRNA) LINC01561, which is transcriptionally upregulated by SOX2, serves as a molecular decoy for miR-760, thereby stabilizing SHCBP1 mRNA and facilitating pro-tumorigenic activities [37]. Another similar regulatory network involves the lncRNA TP53TG1 interacting with miR-33b in retinoblastoma. TP53TG1-mediated sequestration of miR-33b leads to enhanced SHCBP1 expression [38]. These complex post-transcriptional mechanisms permit the rapid modulation of SHCBP1 levels according to urgent cellular needs, particularly during cancer progression.

### 2.4. Post-Translational Modifications of SHCBP1

SHCBP1 is also strongly regulated by a range of post-translational modifications that influence its activity, subcellular distribution, and protein stability (Figure 1D). During mitosis, phosphorylation of SHCBP1 at Ser634 by Aurora kinase B (AURKB) is required for its correct localization to the midbody, while phosphorylation mediated by CDK1 ensures the timely execution of its mitotic roles [6]. In growth factor signaling, EGF-induced activation of HER2 triggers SHCBP1 phosphorylation at Ser273, which is essential for its subsequent nuclear import and for the activation of downstream targets including PLK1 in gastric and bladder cancer cells [23,39]. Importantly, mutation of this phosphorylation site to alanine impedes nuclear translocation and enhances cellular sensitivity to trastuzumab, revealing possible therapeutic implications.

The level of SHCBP1 protein within the cell is also modulated by ubiquitin-mediated proteasomal degradation. In neural progenitor cells, the E3 ubiquitin ligase mLin41 mediates SHCBP1 ubiquitination, stabilizing it in response to fibroblast growth factor (FGF) signaling and supporting neural development [8]. Similarly, TRIM71 regulates SHCBP1 protein stability to facilitate FGF/ERK signaling in embryonic stem cells [40]. The interaction between lncRNA Trincr1 and TRIM71 fine-tunes stem cell differentiation and self-renewal by reducing SHCBP1 protein levels. In esophagogastric junction adenocarcinoma, however, SHCBP1 stability increases through deubiquitination via ubiquitin-specific peptidase 49 (USP49). The stabilized SHCBP1 protein subsequently activates β-catenin signaling, which elevates glutathione peroxidase 4 expression and enhances cancer cell survival under oxidative stress [41]. Detailed characterization of the specific ubiquitination sites and the functional ubiquitin ligases acting on SHCBP1 remains to be studied.

The collective studies establish that SHCBP1 serves as a dynamically modulated adaptor protein performing diverse functions in oncogenic signaling, cell cycle control, and gene expression regulation. Its recurrent overexpression and activation in various human cancers emphasize its role as an important regulator in tumorigenesis. The intricate multi-level regulation of SHCBP1—spanning transcriptional, post-transcriptional, and post-translational processes—demonstrates the need for further investigation into SHCBP1–driven oncogenic mechanisms within specific cellular contexts. Elucidating these regulatory circuits could provide new therapeutic strategies by enabling targeted intervention in SHCBP1 or its downstream effectors in cancer.

## 3. Multifaceted Roles of SHCBP1 in the Hallmarks of Cancer

**Figure 2:**
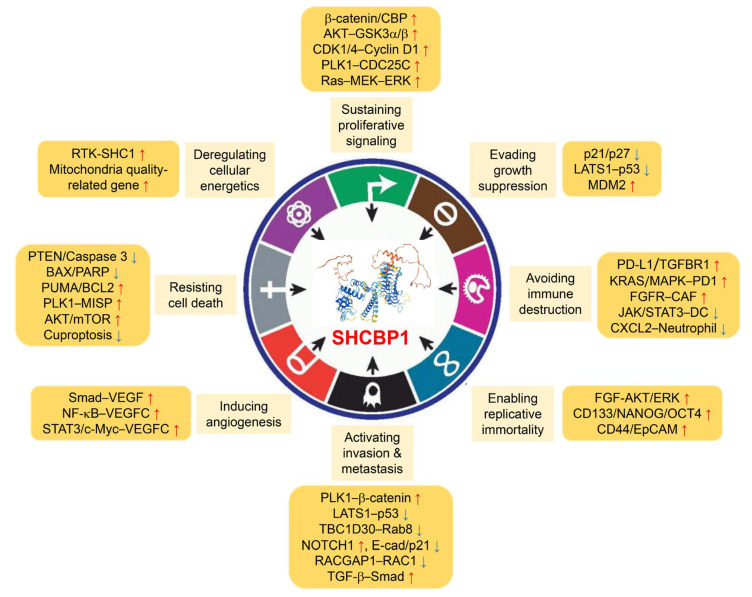
Multifaceted functions of SHCBP1 in cancer hallmarks. Eight defining features of cancers are presented in the light-yellow box, and SHCBP1’s oncogenic contribution to each hallmark is outlined in the dark yellow box. The red arrow represents upregulation or activation of signaling pathways, while the blue arrow indicates inhibition of gene expression or pathways mediated by SHCBP1. (Adapted from [4]).

As summarized in the preceding sections, SHCBP1 functions as a dynamically regulated adaptor protein integrating mitogenic signals, cytoskeletal rearrangements, and transcriptional regulation. Its elevated expression across diverse cancer types, as well as its regulation at transcriptional, post-transcriptional, and post-translational steps, positions SHCBP1 as a potential modulator of various aspects of cancer biology. Emerging evidence indicates that SHCBP1 drives several hallmark features of cancer, including sustained proliferation, apoptotic resistance, invasion, metastasis, and resistance to therapy. Here, we analyze the mechanistic functions of SHCBP1 in these processes and examine its involvement in the canonical hallmarks of cancer (Figure 2).

### 3.1. Sustaining Proliferative Signaling by SHCBP1

Persistent cell proliferation is a hallmark of cancer, arising from continual activation of mitogenic pathways and evasion of cell cycle checkpoints [3]. In normal physiology, cellular proliferation is subject to tight regulation by mitogenic cues, contact inhibition, and robust checkpoint controls. Conversely, cancer cells often circumvent these regulatory mechanisms through the activation of oncogenic pathways, enabling relentless proliferation that does not require external growth factors. This uncontrolled mitogenic signaling represents the underlying molecular basis by which adaptor proteins and cell cycle regulators, including SHCBP1, promote the oncogenic phenotype.

In NSCLC, SHCBP1 augments EGF-driven nuclear β-catenin activity by associating with CREB binding protein (CBP), leading to increased transcription of genes associated with proliferation [21]. Inhibition of the CBP/β-catenin complex by ICG-001 diminishes SHCBP1-dependent cell proliferation and tumor expansion. Furthermore, SHCBP1 collaborates with FGF13 to activate the AKT–GSK3α/β signaling axis, thereby promoting cell cycle progression [42]. Supporting findings from aggressive gastric cancer clinical samples with elevated SHCBP1, knockdown of SHCBP1 in gastric cancer cells suppresses the expression of CDK1, CDK4, and cyclin D1, consistent with a block at the G1/S phase transition and reduced cell proliferation [15].

At the mitotic level, SHCBP1 promotes cell division by interacting with midbody components MKLP1 and MgcRacGAP and activating PLK1 in a phosphorylation-dependent manner [9]. In prostate cancer, SHCBP1 enhances cell proliferation and the G2/M transition via PLK1-mediated activation of CDC25C phosphorylation [43]. The absence of SHCBP1 impairs mitotic progression and leads to decreased levels of cyclin D1 and phosphorylated ERK1/2, which emphasizes its role in maintaining chromosomal stability in HCC [16]. Moreover, SHCBP1 promotes cell growth through activation of the Ras–MEK–ERK cascade, enhancing proliferation and migration signaling pathways in melanoma [44]. Collectively, these findings demonstrate that SHCBP1 is an important mediator of mitogenic and cell cycle-related signaling, thereby supporting the proliferative potential of tumor cells.

### 3.2. Evading Growth Suppression by SHCBP1

In multicellular organisms, cell proliferation is stringently regulated by intrinsic tumor suppressor mechanisms that preserve tissue homeostasis and prevent neoplastic transformation [45]. Interruption of these growth-regulatory pathways constitutes a hallmark of cancer that allows tumor cells to circumvent anti-proliferative controls. Such evasion generates conditions conducive to oncogenic signaling, enabling tumor cells to operate outside of physiological limits and to promote tumor development. Recent studies indicate that, although SHCBP1 is not a traditional oncogene, it plays a significant role in antagonizing tumor-suppressive mechanisms, ultimately conferring a proliferative benefit to cancer cells.

In breast cancer, upregulation of SHCBP1 correlates with suppression of CDKN1A (p21) and CDKN1B (p27), which are crucial inhibitors of CDK activity [13,46]. Knockdown of SHCBP1 results in increased nuclear localization of p21 and p27, suggesting that SHCBP1 impedes their activity either at the transcriptional level or by promoting their degradation. In lung cancer, SHCBP1 also interacts with FGF13 to downregulate p21/p27, thereby allowing cells to bypass the G1 checkpoint [42]. SHCBP1 further contributes to proteasome-mediated degradation of tumor suppressors, including LATS1 and p53, potentially via the MDM2–TP53 pathway in prostate cancer [47]. Together, these data identify SHCBP1 as an important mediator of diverse tumor suppressor networks, enabling cancer cells to advance by simultaneously inhibiting multiple growth-control mechanisms.

### 3.3. Resisting Cell Death by SHCBP1

Apoptosis, also known as programmed cell death, is a crucial biological process that ensures tissue homeostasis by removing damaged, senescent, or potentially dangerous cells. In cancer, the ability to evade apoptosis is a key hallmark that enables cancer cells to survive in the presence of genomic instability, oncogenic stress, or therapeutic interventions [48]. Accumulating evidence indicates that SHCBP1 is involved in promoting apoptotic resistance in various cancer types.

In lung cancer, SHCBP1 downregulates PTEN and inhibits caspase-3 activity, thereby suppressing intrinsic apoptosis [14]. Depletion of SHCBP1 leads to an upregulation of pro-apoptotic proteins (BAX, cleaved caspase-3, and PARP) and a decrease in anti-apoptotic BCL2 expression in gastric cancer [15]. Comparable patterns have been reported in breast cancer, where SHCBP1 modulates the levels of PUMA and BCL2 to enhance cell survival [13]. In HCC, SHCBP1 is implicated in regulating apoptosis through activation of JAK2/STAT3 phosphorylation [49]. Disruption of SHCBP1’s anti-apoptotic function, particularly via interaction with NUSAP1, augments immune cell-mediated cell death by influencing BAX and BCL2 expression.

Furthermore, SHCBP1 is implicated in promoting chemoresistance by suppressing apoptosis-inducing signaling pathways. In gastric cancer, SHCBP1 facilitates cancer cell mitosis through hyperactivation of the HER2-mediated signaling pathway [39]. Upon growth factor stimulation, SHCBP1 interacts with PLK1 to enhance phosphorylation of MISP, resulting in increased cell survival. Inhibition of the SHCBP1 and PLK1 interaction sensitizes gastric cancer cells to trastuzumab treatment. SHCBP1 also fosters cisplatin resistance through activation of Wnt/β-catenin signaling in head-and-neck squamous cell carcinoma (HNSCC) and lung cancer [50,51]. Recent findings reveal that SHCBP1 also suppresses autophagic cell death by activating the AKT/mTOR pathway in ovarian cancer [52]. Combined targeting of SHCBP1 and use of an autophagy inhibitor sensitizes ovarian cancer cells to cisplatin treatment. Collectively, these findings indicate that SHCBP1 contributes to cellular survival not only under basal conditions but also by protecting tumor cells against cytotoxic therapies.

As a recently characterized cell death mechanism, the cuproptosis pathway influences tumor growth by disrupting mitochondrial function upon copper exposure and inducing proteotoxic stress [53]. Multiple cuproptosis-related genes have emerged as potential prognostic markers in cancer, owing to their involvement in cancer progression, drug responsiveness, and patient clinical characteristics. Notably, SHCBP1 is identified as a cuproptosis-related gene through bioinformatic analysis in esophageal adenocarcinoma [54]. Copper treatment targeting SHCBP1 enhances cell death and copper accumulation, while also increasing the expression of copper import genes. Collectively, these findings reveal a new aspect of SHCBP1 function in regulating metabolic stress-mediated cell death.

### 3.4. Enabling Replicative Immortality by SHCBP1

Cancer stemness represents a critical hallmark of tumor progression, as it provides cancer cells with the capacity for self-renewal, differentiation plasticity, and replicative immortality, thereby promoting tumor heterogeneity and therapeutic resistance [55]. Preliminary study suggests that SHCBP1 contributes to these processes by sustaining stem cell-like properties and regulating transcriptional programs linked to pluripotency.

In neural progenitor cells, SHCBP1 stability is maintained through E3 ubiquitin ligase mLin41-mediated ubiquitination in an FGF-dependent context, resulting in activation of downstream AKT/ERK signaling pathways that preserve self-renewal and proliferative potential [8]. SHCBP1’s involvement in stem cell maintenance is also confirmed via TRIM71-mediated FGF/ERK signaling activation [40]. In cervical cancer, SHCBP1 critically contributes to the cancer stem cell-like phenotype by regulating the expression of stemness-linked genes such as CD44, CD133, NANOG, and OCT4 [56]. SHCBP1 enhances these stem cell markers through EIF5A-dependent nuclear translocation of the NF-κB transcription factor. Additionally, EGF stimulation promotes SHCBP1 nuclear localization, where it interacts with CBP to facilitate β-catenin acetylation, thereby activating stem cell markers including CD44 and EpCAM in NSCLC [21]. Altogether, these findings indicate that SHCBP1 may support the persistence of stem-like, therapy-resistant populations, facilitating sustained tumor growth and recurrence.

### 3.5. Inducing Angiogenesis by SHCBP1

Angiogenesis, the formation of new blood vessels from existing vasculature, is critical for tumor growth beyond a certain threshold and for metastatic spread. This process relies on a precisely regulated equilibrium between pro-angiogenic and anti-angiogenic mediators, with vascular endothelial growth factor (VEGF), angiopoietins, FGFs, and hypoxia-inducible factors serving as principal activators [57]. While canonical angiogenic pathways have been comprehensively studied, recent data indicates that non-canonical signaling proteins, including those not previously classified as angiogenic regulators, can modulate this process indirectly.

Although SHCBP1 is not yet firmly recognized as a pro-angiogenic protein, accumulating data support its potential role in angiogenesis-related signaling pathways. In synovial sarcoma, upregulation of SHCBP1 resulted in greater VEGF secretion by cancer cells, which enhanced HUVEC tube formation and migration in vitro [22]. The observed phenotype was associated with TGF-β1-driven EMT, where increased expression of SHCBP1 accompanies the transition to a mesenchymal state. In glioma, higher SHCBP1 expression was linked to elevated VEGFC levels, and functional enrichment analysis revealed participation in NF-κB–regulated pro-angiogenic gene networks [19]. Evidence further demonstrates that SHCBP1 modulates the angiogenic pathway through regulation of VEGFC expression via STAT3/c-Myc signaling in penile cancer [58]. Collectively, these findings indicate that SHCBP1 influences VEGF family gene regulation through inflammatory mediators or EMT-related transcription factors, rather than by directly acting on traditional angiogenic receptors. Furthermore, as the RTK binding protein Shc1 is established to mediate angiogenesis via VEGF-A-dependent signaling [59] and SHCBP1 interacts with phosphorylated SHC1, the SHC–SHCBP1 axis may indirectly facilitate vascular remodeling in tumor contexts. Although additional mechanistic investigations are required, these results imply that SHCBP1 could contribute to angiogenesis through RTK-dependent signaling pathways in a context-specific fashion.

### 3.6. Activating Invasion and Metastasis by SHCBP1

Metastasis, the leading cause of cancer-related death, encompasses several steps process involving EMT, local invasion, intravasation, survival within the circulatory system, and colonization of distant tissues [60]. A pivotal factor in metastasis is the acquisition of mesenchymal traits by epithelial tumor cells, which enables their detachment and migration through extracellular barriers. EMT is orchestrated by key transcription factors and is controlled by pathways such as TGF-β, PI3K/AKT, Wnt/β-catenin, and MAPK.

SHCBP1 is consistently associated with the promotion of EMT and metastatic characteristics in multiple cancer types [20,61]. In metastatic prostate cancer, SHCBP1 expression is significantly upregulated and is linked to later disease stages and decreased survival outcomes [43]. Functional investigations reveal that SHCBP1 facilitates PLK1-mediated phosphorylation events, which are critical for driving EMT and bone metastasis. Moreover, SHCBP1 accelerates the degradation of essential tumor suppressors, such as LATS1 and p53, thereby amplifying metastatic behavior by inhibiting the Hippo pathway and p53 axis [47]. SHCBP1 also modulates cytoskeletal organization, a process vital for tumor cell migration and invasion. In unciliated ductal carcinomas, SHCBP1 forms a complex with GTP hydrolysis-activating protein (TBC1D30) to suppress Rab8 GTPase activity, thereby impeding the ciliogenesis process and augmenting migratory capabilities [62]. Additionally, in pancreatic ductal adenocarcinoma (PDAC), SHCBP1 is involved in NOTCH1 O-glycosylation through EGF domain specific O-linked GlcNActransferase (EOGT), which leads to enhanced nuclear localization of the Notch intracellular domain and promotes the expression of mesenchymal markers while suppressing the transcriptional activity of E-cadherin and p21 [63]. In bladder cancer, SHCBP1 augments EGF-induced invasive behavior by associating with RACGAP1 and restricting the GTP hydrolysis activity of RAC1, a critical regulator of actin cytoskeleton dynamics and cell motility [23]. The involvement of SHCBP1 in TGF-β1-induced EMT is also validated in synovial sarcoma, where SHCBP1 is upregulated following TGF-β1 stimulation [22]. In glioma, elevated SHCBP1 levels are associated with invasive gene signatures and a poorer prognosis, and its influence on NF-κB signaling further highlights its contribution to tumor spread [19]. Collectively, these findings identify SHCBP1 as an important regulator of EMT, cytoskeletal restructuring, and metastatic signaling.

### 3.7. Deregulating Cellular Energetics by SHCBP1

Metabolic reprogramming is an essential hallmark of cancer, allowing cancer cells to maintain uncontrolled growth and survive in environments with limited nutrients or low oxygen levels [64]. A well-recognized metabolic adaptation is the Warburg effect, where cancer cells preferentially use aerobic glycolysis over oxidative phosphorylation even when oxygen supply is adequate. This metabolic shift not only generates ATP rapidly but also facilitates anabolic processes required for cellular growth.

Although direct functional evidence linking SHCBP1 to metabolic reprogramming is still lacking, several indirect associations point to its potential involvement. For instance, ShcA, which interacts with SHCBP1, has been demonstrated to enhance both glycolysis and mitochondrial respiration in breast cancer cells through RTK-dependent activation and upregulation of PGC-1α, a central regulator of mitochondrial biogenesis [65]. Given SHCBP1’s association with SHC1 and RTK pathways, it remains plausible that SHCBP1 modulates cellular metabolism. Transcriptomic studies in lung adenocarcinoma have classified SHCBP1 within a mitochondrial quality-related gene (MORG) signature, where its high expression is linked to unfavorable prognosis and possible disruptions in mitochondrial homeostasis [66]. These findings suggest that SHCBP1 could influence energy metabolism by altering mitochondrial dynamics or biogenesis. However, delineating the precise molecular mechanisms by which SHCBP1 regulates cellular metabolic processes is an outstanding challenge. Further investigations using metabolic flux analysis, mitochondrial stress tests, and isotope tracing will be essential to determine whether SHCBP1 directly contributes to metabolic reprogramming in cancers.

### 3.8. Avoiding Immune Destruction by SHCBP1

Immune evasion by cancers constitutes a great barrier to successful anti-cancer immune responses. Under physiological conditions, damaged cells are targeted and eliminated through immune surveillance mediated by cytotoxic T lymphocytes, natural killer cells, and antigen-presenting cells. Cancers, however, employ diverse mechanisms to evade immune detection, such as the upregulation of immune checkpoint molecules, increased secretion of immunosuppressive cytokines, and restructuring of the tumor microenvironment (TME) to promote regulatory and suppressive immune subsets [67,68].

Recent pan-cancer analyses identified SHCBP1 as an important factor in immune evasion. High SHCBP1 expression is linked to greater infiltration of tumor-associated macrophage (TAM), CD4^+^ naïve T cells, CD8^+^ T cells, and neutrophils, as well as elevated levels of immune checkpoint proteins, including PD-L1 and TGFBR1 in multiple tumor types [31,69]. These associations indicate that SHCBP1 may influence the immune landscape of the TME by affecting tumor mutation burden (TMB) and microsatellite instability (MSI) status. In breast cancer, SHCBP1 also modulates the immunosuppressive TME by upregulating immune checkpoint gene expression and changing patterns of immune cell infiltration [30]. At the functional level, increased SHCBP1/SMYD3 in BRCA1–deficient cells drive the activation of KRAS/MAPK signaling, resulting in enhanced PD1 expression in T cells and the development of immunotherapy resistance. Notably, SHCBP1 knockout in mice improves the efficacy of combination therapy with Erdafitinib and anti-PD1 antibody in breast cancer models [24]. These findings imply that SHCBP1 facilitates immune escape via FGFR-dependent enhancement of α-smooth muscle actin positive cancer–associated fibroblast (CAF) infiltration and suppression of cytotoxic T cell infiltration. SHCBP1 is additionally implicated in JAK2/STAT3–driven dendritic cell differentiation through increased IL-6 secretion in HCC [49]. The effect of SHCBP1 on dendritic cell maturation impacts tumor immunity and influences sensitivity to immune checkpoint inhibitor (ICI) therapy. Furthermore, SHCBP1 downregulates the expression of CXCL2, a chemokine important for neutrophil recruitment and resolution of inflammation, thereby supporting tumor cell proliferation through activation of AKT and ERK signaling and suppression of p21 and p27 [46]. However, whether SHCBP1 directly modulates cytokine gene transcription or acts via epigenetic or signaling pathways is yet to be determined.

## 4. SHCBP1 as a Diagnostic and Prognostic Biomarker in Cancer

The diverse oncogenic roles of SHCBP1, as discussed in previous sections, highlight its utility as a potential biomarker for multiple cancers. Increasing evidence from transcriptomic and proteomic analyses in various cancers indicates that elevated SHCBP1 expression is strongly linked to aggressive clinicopathological traits and poor patient prognoses (Table 1). This section reviews data on the clinical significance of SHCBP1 expression, utilizing public cancer databases, bioinformatic results, and analyses of patient tissue specimens.

In breast cancer, both SHCBP1 mRNA and protein levels are significantly higher in tumor tissues when compared to normal tissue counterparts. Detailed investigation of TCGA datasets indicates that elevated SHCBP1 expression is strongly correlated with advanced clinicopathological characteristics, such as increased tumor size, higher histological grade, and lymph node metastasis [13,46]. Immunohistochemical analyses further validate that patients with elevated SHCBP1 experience significantly shorter overall survival and higher relapse rates. Importantly, SHCBP1 is commonly identified as part of multi-gene prognostic signatures for breast cancer patients [70,71,72,73]. For example, a four-gene panel (DOK4/HCCS/PGF/SHCBP1) reliably stratifies patients by metastasis risk in external validation cohorts, and machine learning models that include SHCBP1 can accurately estimate TMB and stemness indices. Another investigation utilized a machine learning algorithm to identify SHCBP1 as one of the multi-gene signatures for the cancer stem cell index (mRNAsi) in breast cancer, demonstrating a strong association with clinicopathological features, including pathological stage, metastasis, and overall survival [74]. Incorporation of SHCBP1 into ceRNA and lactylation–associated regulatory networks has further enhanced its value for identifying subgroups of high-risk breast cancer patients who are less responsive to therapy [75,76]. As SHCBP1 is repeatedly recognized as a key component in multi-gene prognostic models in several studies, its expression may function as a reliable biomarker for differentiating breast cancer patient risk and informing personalized treatment plans.

Studies in PDAC highlight the diagnostic value of SHCBP1, demonstrating that tissue expression can distinguish cancers from benign pancreatic lesions with high sensitivity and specificity [63,77]. Higher SHCBP1 levels are associated with more aggressive clinicopathological features, including portal vein invasion and advanced TNM stage, contributing to markedly shorter overall survival, disease-free survival, and progression-free interval. SHCBP1 also exhibited good diagnostic accuracy for PDAC, with its expression levels corresponding closely to serum CA19-9 concentrations. Transcriptomic profiling and protein–protein interaction (PPI) network analyses have consistently identified SHCBP1 as one of 12 hub genes implicated in poor prognosis and resistance to therapy among PDAC patient samples [78].

In lung cancer, including NSCLC and small cell lung cancer (SCLC), SHCBP1 is identified as a key component within gene networks implicated in brain metastasis, immune evasion, and metabolic regulation [66,79,80,81]. SHCBP1 in lung adenocarcinoma is significantly upregulated in metastatic lesions, showing a strong association with unfavorable drug response and reduced overall survival among patients. Analysis using circular RNA and PPI network-based approaches has pinpointed SHCBP1 as one of five central hub gene signatures, which are closely correlated with patient overall survival [82]. In addition, SHCBP1 is included in CAF–related gene signatures that serve as predictors of immunotherapy response and TME remodeling [83]. Elevated SHCBP1 expression within CAF gene signatures demonstrates significant associations with pathological characteristics, influencing immune cell infiltration and cytokine expression.

In HCC, increased SHCBP1 expression, in combination with noncoding RNA levels, is associated with activation of mitotic genes and poorer survival outcomes in both TCGA and GEO cohorts [84,85]. Elevation of SHCBP1 is confirmed in cancer tissue relative to matched normal controls and is markedly linked to adverse patient survival. Another investigation identified SHCBP1 as one of 11 hub genes contributing to cancer progression and prognosis through comprehensive PPI network and functional enrichment analysis [86]. Follow-up analysis further corroborated that high SHCBP1 levels in HCC specimens are associated with decreased patient survival rates. Importantly, SHCBP1 overexpression is also observed in hepatitis B virus–related HCC and shows an association with poor survival, although detailed relationships with clinical indicators require additional clarification [87,88].

In prostate cancer, SHCBP1 expression is strongly associated with key clinical variables, such as PSA titers, Gleason score, seminal vesicle invasion, and disease stage [47]. More advanced tumors manifest substantially worse recurrence-free survival when SHCBP1 is highly expressed. Additionally, SHCBP1 expression displays a strong relationship with elevated T stage, higher Gleason scores, augmented propensity for bone metastasis, and diminished progression-free survival [43]. Machine learning-based classification tools, including PRADclass, have identified SHCBP1 as a marker specific to Gleason grade 5 and validated this association across separate datasets [89]. The robustness of PRADclass and its signature gene set is supported by validation with random datasets, highlighting its potential utility in distinguishing indolent from aggressive prostate cancer.

Beyond these major tumor types, SHCBP1 also exhibits significant prognostic potential in additional cancers. For instance, elevated SHCBP1 expression is observed in glioblastoma when compared to normal brain tissues and is linked to poorer overall survival rates in patients [19]. In a parallel study, SHCBP1 was identified as a prognostic marker for glioma, demonstrating a strong predictive value based on the PART1–miRNA-429–SHCBP1 axis [36]. The utilization of an advanced analysis method, WGCNA, further recognized SHCBP1 as one of the prognostic markers, showing a substantial correlation with tumor stage and diminished survival [90]. To elucidate differences in treatment outcomes between male and female glioblastoma patients, the researchers examined sex-specific transcriptome data and PPI network profiles [91]. Notably, SHCBP1 is identified as one of the key genes upregulated in female tumors and associated with poorer patient survival rates. Additionally, SHCBP1’s involvement as both a diagnostic and prognostic marker is reported in a range of other cancers, including esophageal cancer, retinoblastoma, synovial sarcoma, HNSCC, ovarian cancer, and gastric cancer, all showing high correlation scores [17,22,38,50,52,54,92].

According to multiple pan-cancer analyses, SHCBP1 expression is also positively associated with TMB, MSI, immune cell infiltration values, and the expression of immunosuppression-related genes, such as TGFBR1, PD-L1, and TGFB1 in various cancers [31,69,93]. Moreover, increased SHCBP1 expression is significantly linked to reduced overall survival, likely via alteration of the immunosuppressive TME in most cancers. In addition, analysis using the Genomics of Drug Sensitivity in Cancer (GDSC) database indicates that elevated SHCBP1 in patients contributes to resistance against multiple chemotherapies [31]. Taken together, these observations indicate that SHCBP1 may serves not only as a surrogate marker for tumor aggressiveness, but also as an integrative indicator of molecular subtype, immune evasion, and therapeutic response. Incorporation of SHCBP1 into diagnostic panels, prognostic models, or therapeutic decision-making frameworks could help guide patient treatment.

**Table 1:** SHCBP1 expression levels and clinical relevance across various cancer types.

Cancer Type	Relevant Clinical Characteristics	Prognostic Significance and Reference
Breast cancer	Tumor size ↑, Grade ↑, Lymph node metastasis ↑, TMB ↑, Stemness index ↑	OS ↓ *p* < 0.001 [13], *p* < 0.01 [72], HR = 1.59 [46], HR = 1.76 [73], HR = 1.06 [74]; RFS ↓ *p* < 0.001 [13],
Pancreatic cancer	Tumor size ↑, Portal vein invasion ↑, AJCC stage ↑, CA19-9 ↑	OS ↓ *p* < 0.001 [63], HR = 1.57 [77], HR = 1.6 [78]; DFS ↓ *p* < 0.001 [63], HR = 1.8 [77]; PFI ↓ HR = 1.57 [77]
Lung cancer	TNM stage ↑, Metastasis ↑, Immune evasion ↑, TMB ↑	OS ↓ *p* < 0.001 [66], *p* = 0.0135 [79], *p* = 0.047 [80], *p* < 0.001 [81], HR = 1.5 [82]
HCC	SHCBP1 level ↑, Immune cell infiltration ↑, TPX2 ↑	OS ↓ *p* < 0.001, HR = 2.14 [84], HR = 1.5 [85], HR = 1.5 [86], HR = 1.83 [88]
Prostate cancer	PSA ↑, Gleason grade ↑, pT stage ↑, Seminal vesicle invasion ↑	PFS ↓ *p* < 0.001 [43]; RFS ↓ *p* < 0.001 [47]
Glioblastoma	pT stage ↑, IDH mutation status ↑, Female tumor ↑	OS ↓ *p* < 0.01 [19], *p* < 0.001 [36], *p* = 0.039 [90]
Pan-cancer	TMB ↑, MSI ↑, TAM ↑, Immune evasion ↑, IC50 ↑	OS ↓ *p* < 0.001 [31], HR = 1.29–1.8 [69], HR = 1.764 [93]; DFS ↓ *p* < 0.05 [69], HR = 1.469 [93]; PFI ↓ *p* < 0.001 [31], HR = 1.661 [93]

↑ represents an increase in the indicated phenomenon, whereas ↓ represents a decrease. Abbreviations: OS, Overall survival; HR, Hazard ratio; RFS, Relapse-free survival; TMB, Tumor mutation burden; AJCC, American joint committee on cancer; CA19-9, Cancer antigen 19-9; DFS, Disease-free survival; PFI, Progression-free interval; HCC, Hepatocellular carcinoma; TPX2, Targeting Protein for Xenopus kinesin-like protein 2; PSA, Prostate antigen; PFS, Progression-free survival; IDH, Isocitrate dehydrogenase; MSI, Microsatellite instability; TAM, Tumor-associated macrophage; IC50, half-maximum inhibitory concentration.

## 5. Therapeutic Targeting of SHCBP1 in Cancer

Considering the diverse roles of SHCBP1 in tumor progression, therapy resistance, and modulation of the TME, it has been recognized as an attractive therapeutic target. While no SHCBP1-specific inhibitors have yet been implemented clinically, preclinical investigations utilizing genetic and pharmacological interventions have demonstrated that targeting SHCBP1 can suppress tumor growth and improve treatment outcomes. This section reviews present strategies for SHCBP1 inhibition, including RNA interference (RNAi), CRISPR-Cas9, and pharmacologic compounds, and discusses combination approaches that leverage SHCBP1’s diverse function in oncogenic networks (Table 2).

### 5.1. Genetic Suppression of SHCBP1 for Antitumor Therapy

For several cancers, small interfering RNA (siRNA)-mediated SHCBP1 knockdown diminishes tumor cell proliferation, induces apoptosis, and suppresses metastatic capabilities. In lung cancer, SHCBP1 silencing induces caspase-3 activation through PTEN upregulation and suppresses Wnt/β-catenin-mediated survival signaling [14,51]. Comparable findings have been observed in breast cancer cells, where SHCBP1 knockdown led to ERK1/2 pathway inhibition and CXCL2 upregulation, contributing to decreased colony formation and cell viability [46]. Functional loss studies using shRNA against SHCBP1 indicate EIF5A-dependent NF-κB activation is necessary for cervical cancer cell proliferation and self-renewal [56]. More broadly, SHCBP1 depletion by siRNA or shRNA uniformly disrupts cell cycle progression, increases apoptosis, and attenuates invasiveness across multiple cancer models [15,43,50,63].

Moving beyond transient gene silencing, CRISPR-Cas9-mediated genome editing produced more sustained antitumor effects. In breast and pancreatic cancer models, SHCBP1 knockout suppressed tumor progression in both 2D and 3D organoid systems and in patient-derived xenograft models [13,77]. Similarly, conditional SHCBP1 deletion in mammary epithelial cells using MMTV-Cre transgenic mice markedly slowed breast cancer development and metastasis through restored Rab8-mediated ciliogenesis [62]. Notably, CRISPR-based ablation of SHCBP1 in CAFs improved the therapeutic response to Erdafitinib and αPD-1 co-treatment by promoting T cell infiltration and disrupting the immunosuppressive TME [24].

Overall, these findings establish SHCBP1 as a therapeutically actionable target whose genetic suppression may enhance the effectiveness of standard chemotherapy and immunotherapy strategies across a broad spectrum of cancers. Advances in nanoparticle-mediated delivery systems, including liposomes and micelles, have improved the stability and tumor-specific delivery of RNAi molecules in vivo [94,95,96]. Clinical investigations targeting oncogenic drivers using novel delivery systems validated favorable safety profiles and efficacy in early-phase trials, thereby reinforcing the potential for SHCBP1-targeted RNAi therapy [97,98].

### 5.2. Pharmacological Inhibition of SHCBP1 for Anticancer Therapy

Pharmacologic inhibition of SHCBP1 remains difficult due to the lack of enzymatic domains or clearly defined active sites. However, computational screening, structure-based drug design, and transcriptome-guided compound selection have led to the identification of several candidate molecules that target SHCBP1 or its related signaling pathways.

Structure-based virtual screening using the AlphaFold-predicted SHCBP1 model identified AZD5582 as a potential SHCBP1-binding compound, which can suppress pancreatic cancer growth and metastasis through induction of apoptosis [77]. A different approach integrating transcriptome data with a candidate drug database identified rucaparib, a PARP inhibitor, as a putative SHCBP1 inhibitor [81]. Rucaparib decreased cell proliferation and metastatic potential in lung adenocarcinoma cells by inhibiting SHCBP1 at the transcriptional level. Using a similar in silico methodology, arachidonyltrifluoromethane was identified as a putative SHCBP1-interacting compound through CMap analysis in breast cancer [76]. Another bioinformatic analysis highlighted several candidate drugs that may target SHCBP1-associated pathways involved in HCC progression [85]. Validation of these drug–SHCBP1 interactions and assessments of their therapeutic efficacy in cancer growth remains necessary using in vitro and in vivo models in future studies.

Recently, numerous pharmaceutical companies have focused on identifying anti-tumorigenic compounds from natural products by employing enhanced analytical tools and rational strategy development [99,100]. Theaflavin-3,3′-digallate (TFBG), a polyphenolic compound derived from black tea, was discovered as a specific inhibitor of the SHCBP1–PLK1 interaction through virtual screening [39]. TFBG showed strong affinity for the PLK1 binding pocket, thereby blocking SHCBP1 and PLK1 association, which led to cancer growth inhibition and enhanced sensitivity to trastuzumab resistance in gastric cancer. Resveratrol, a well-established anti-cancer agent, markedly inhibited cell proliferation, colony formation, and invasion of melanoma cells [44]. Mechanistic investigation revealed that resveratrol suppressed SHCBP1 transcription and reduced downstream ERK1/2 phosphorylation, thus affecting cell proliferation-related signaling pathways. In a separate study, Ginsenoside Rh7, a protopanaxatriol-type saponin obtained from ginseng, was found to inhibit cell proliferation, migration, and invasion by blocking SHCBP1-mediated β-catenin nuclear localization in gastric cancer [92]. This compound exhibited anti-cancer effects by downregulating SHCBP1 mRNA expression and subsequently diminishing the interaction between SHCBP1 and β-catenin.

These findings indicate that SHCBP1-targeted small molecules, whether acting directly or through its downstream pathways, offer a promising therapeutic strategy, especially in cancers exhibiting high SHCBP1 expression.

### 5.3. SHCBP1-Targeted Combination Strategies in Cancer Therapy

The important role of SHCBP1 in various oncogenic and immunomodulatory pathways highlights its value as a sensitizing target for combination cancer therapies. For example, ICG-001 substantially inhibited EGF or SHCBP1-mediated CBP/β-catenin interaction in lung cancer [21]. In ovarian cancer, SHCBP1 overexpression induces cisplatin resistance by activating AKT/mTOR signaling and inhibiting autophagic cell death. Combined treatment with SHCBP1 siRNA and autophagy inhibitors reverses cisplatin resistance and decreases tumor burden in vivo [52]. Additionally, SHCBP1 depletion potentiated the sensitivity of lung cancer cells to low dose genotoxic agents, such as cisplatin, etoposide, and radiation, by facilitating mitotic catastrophe through G2/M checkpoint abrogation [29]. Notably, targeting SHCBP1 within the TME improves the efficacy of combination immunotherapies. In breast cancer models, CAF–specific SHCBP1 knockout enhanced the antitumor activity of Erdafitinib and PD-1 blockade, largely by influencing immune cell recruitment and inhibiting CAF activity [24]. Similarly, combined administration of trametinib and αPD-1 antibody inhibited SMYD3/SHCBP1-dependent breast cancer progression in xenografts by suppressing MEK activation and promoting CD4/8+ cell infiltration [30]. Collectively, these findings are consistent with pan-cancer analyses indicating SHCBP1 as a contributor to immune evasion and therapeutic resistance.

Taken together, SHCBP1 emerges as a compelling, yet inherently challenging, therapeutic target across diverse cancers. Its inhibition not only disrupts cancer-intrinsic growth and survival pathways, but also enhances the efficacy of combination therapies such as chemotherapy and immunotherapy, thereby raising the possibility of its application into clinical trials. However, the lack of enzymatic or ligand-binding domains makes the direct pharmacological targeting of SHCBP1 intrinsically difficult, and most of the current therapeutic studies remain at an early preclinical stage with limited validation in clinical trials. Furthermore, the translation of these preliminary findings into practice requires a more studies for druggability, biomarker development, and long-term safety, underscoring that significant conceptual and technical hurdles remain to be addressed before SHCBP1 can be considered a realistic therapeutic option.

## 6. Limitations and Future Directions

In this review, we summarized the roles of SHCBP1 in cancer hallmarks, exerting diverse functions in regulating mitotic dynamics, oncogenic signaling, stemness, and metastasis. However, despite these broad functional associations, limitations still remain in fully defining its mechanistic contributions. In particular, while SHCBP1 is implicated in processes such as metabolic reprogramming, stemness, angiogenesis, and immune evasion, current evidence suggests that its influence is often exerted indirectly through modulation of upstream or downstream mediators rather than by direct regulation of these pathways. This distinction raises important questions about the extent to which SHCBP1 serves as a driver versus a passenger of these hallmarks. To address this gap, future studies should aim to provide direct experimental evidence clarifying whether SHCBP1 actively orchestrates these processes in cancer pathology, or whether its role is secondary to broader signaling cascades. Resolving this uncertainty will be essential for accurately defining SHCBP1’s therapeutic relevance and for prioritizing strategies that target its most critical oncogenic functions.

Although previous preclinical evidence from in vitro models, animal studies, and large-scale bioinformatic analyses supports SHCBP1 targeting, the development of potent direct SHCBP1 inhibitors remains technically difficult. As a non-enzymatic scaffold protein, SHCBP1 does not possess a catalytic domain or a well-characterized ligand binding site, limiting the applicability of standard small-molecule inhibitor design. To date, most studies exploring SHCBP1 inhibition remain at a preliminary stage and the observed therapeutic effects are often mediated indirectly through modulation of upstream regulators or downstream effectors rather than through direct targeting SHCBP1 itself [77,81]. These limitations underscore the need for further mechanistic and translational studies to overcome. Encouragingly, recent advances in PPI inhibitors [101,102], molecular glues [103,104], and PROTAC-based protein degraders [105,106] may provide innovative opportunities to target the undruggable SHCBP1 protein.

Another limitation in developing SHCBP1-targeted therapies lies in the incomplete understanding of its three-dimensional structure, which requires for rational drug discovery and the identification of specific inhibitors. To overcome this challenge, precise structural characterization of SHCBP1—particularly its modular domains that mediate interactions with mitogenic and oncogenic partners—is imperative. High-resolution techniques like X-ray crystallography or cryo-EM may delineate critical conformational aspects essential for designing selective and high-affinity inhibitor [107,108]. In addition, recent advances in AI-based protein structure prediction platforms, including AlphaFold3, offer new opportunities to resolve SHCBP1 architecture at near-atomic resolution, thereby accelerating the discovery of novel compounds or enabling the repositioning of existing drugs with potential SHCBP1 specificity [109,110]. Moreover, a thorough analysis of the dynamic and context-dependent SHCBP1 interactome, alongside investigations into its spatial and temporal regulation during cell cycle progression, stress adaptation, and TME-related cues, may reveal new therapeutic avenues and facilitate rational drug discovery.

A further important, though insufficiently investigated, avenue involves SHCBP1’s contribution to the modulation of anti-tumor immunity. Its mechanistic participation in pathways mediating immune evasion has not been clearly defined, and clarifying its role within the immune microenvironment of tumors could support the rationale for combinatorial treatments involving ICIs. Notably, SHCBP1 expression and cellular reliance are highly modulated by tumor context. Large-scale pan-cancer studies suggest associations between SHCBP1 amplification or overexpression and tumor characteristics such as mitotic stress, stemness features, or hyperactive oncogenic signaling [31,72]. Consequently, inferring SHCBP1 dependency patterns through integrated functional genomics and comprehensive transcriptomic analyses will be instrumental in identifying patient cohorts most likely to benefit in future clinical interventions.

Moreover, successfully implementing SHCBP1-targeted therapies in the clinic will rely on the creation and deployment of companion diagnostics capable of robustly quantifying SHCBP1 expression or functional activity in cancer tissues [111,112]. However, there is currently a lack of standardized methods or validated protocols for accurately measuring SHCBP1 in patients, making it difficult to define clear criteria for patient stratification. In addition, because SHCBP1 is also highly expressed in activated lymphocytes and stem cells, it will be important to develop diagnostic strategies that can discriminate between cancer-intrinsic SHCBP1 expression and that originating from immune cells within the TME [5]. The establishment of such refined diagnostic tools is critical for the accurate selection of patients most likely to benefit from SHCBP1-directed strategies. Cancers with elevated SHCBP1 expression or those characterized by resistance to current therapies may be particularly sensitized to these interventions. Furthermore, the use of SHCBP1-targeted inhibitors in rational combination regimens with standard cytotoxic agents, targeted therapies, or immunotherapeutic approaches may ultimately improve survival outcomes for cancer patients.

## 7. Conclusions

SHCBP1 is recognized as a multifunctional adaptor protein of considerable importance in cancer biology. As discussed in this review, SHCBP1 is consistently overexpressed in a variety of cancers and facilitates tumorigenesis by coordinating a broad range of oncogenic activities and modulating the hallmarks of cancer. These activities encompass promoting sustained proliferative signaling, resisting apoptosis, contributing to invasion and metastasis, supporting replicative immortality, and influencing metabolic reprogramming and immune evasion. On a mechanistic level, SHCBP1 functions as an important scaffold that mediates several major oncogenic signaling pathways, such as the EGFR–MAPK, PLK1–CEP55, and PI3K–Akt axes. It undergoes regulation at the transcriptional, epigenetic, and post-transcriptional stages, allowing for context-dependent modulation of its expression and activity. This regulatory flexibility enables SHCBP1 to adapt its function within diverse TMEs and promote tumor aggressiveness.

From a clinical perspective, elevated SHCBP1 expression is associated with poor prognosis, including higher tumor grade, lymphovascular invasion, and diminished overall and disease-free survival across diverse cancer types such as breast cancer, lung adenocarcinoma, HCC, glioblastoma, and gastric cancer. In some scenarios, SHCBP1 is linked to resistance to therapies, especially those involving DNA-damaging agents and immunotherapy.

From a therapeutic standpoint, genetic or pharmacological inhibition of SHCBP1 through RNAi, CRISPR-mediated editing, or modulation of its upstream regulatory factors has produced strong anti-cancer effects in various preclinical models. These observations highlight SHCBP1 as a druggable target, particularly in cancers characterized by mitotic dependence, EMT features, or high proliferation. Notably, suppression of SHCBP1 also augments the anticancer efficacy of conventional chemotherapy and targeted agents, supporting its emerging role in combination therapeutic approaches.

## Figures and Tables

**Table 2:** Preclinical approaches to SHCBP1 inhibition and their therapeutic outcomes.

Targeting Strategy	Therapeutic Agent	Mode of Action	Experimental Model	Therapeutic Effect	Reference
Genetic suppression	siRNA/ shRNA	Transient gene silencing	Cancer cell lines, Xenograft	Inhibition of cellular proliferation, colony formation, self-renewal, and metastasis Induction of apoptosis	[14,15,43,46,50,51,56,63]
	CRISPR-Cas9	Genomic disruption	Cancer cell lines, Xenograft	Decreased tumor progression, suppressed colony formation, and reduced lung metastasis	[13,77]
			Genetically engineered knockout mouse	Attenuated tumor growth and diminished lung metastasis Restoration of ciliogenesis or enhancement of T cell infiltration	[24,62]
Low-molecular-weight compound	AZD5582	SHCBP1-specific inhibitor	Pancreatic cancer cell, organoid model, Xenograft model	Suppression of cellular proliferation and metastatic potential, Induction of apoptotic pathways	[77]
	Rucaparib	PARP enzyme inhibitor	Lung cancer cell line, Xenograft model	Decreased cellular proliferation, inhibition of EMT progression, and suppressed metastasis	[81]
Natural product	TFBG	Inhibitor targeting SHCBP1-PLK1 interaction	Gastric cancer cell, Xenograft model	Suppressed colony formation and tumor development, Enhanced drug responsiveness	[39]
	Resveratrol	SHCBP1 transcriptional repressor	Melanoma cell	Suppression of cellular proliferation, colony formation, and invasive capacity	[44]
	Ginsenoside Rh7	SHCBP1 transcriptional repressor	Gastric cancer cell, Xenograft	Diminished cellular proliferation, colony formation, EMT, and invasiveness	[92]
Combination therapy	siRNA/ ICG-001	Inhibitor of CBP- β-catenin	Lung cancer cell, Xenograft model	Inhibition of sphere formation, stem cell properties, tumor progression, and cell viability	[21]
	siRNA/ Chloroquine	Inhibitor of autophagy	Ovarian cancer cell xenograft	Decreased cellular proliferation within tumors, Enhanced sensitivity to cisplatin	[52]
	shRNA/ Etoposide	DNA-damaging agent	Lung cancer cell, Xenograft model	Suppressed cell cycle progression and diminished tumor growth, Enhanced apoptosis and cellular senescence	[29]
	Erdafitinib/ αPD-1	FGFR inhibitor/ ICI	Conditional SHCBP1 knockout mouse	Suppression of CAF infiltration and breast cancer progression, Enhanced T cell infiltration	[24]
	Trametinib/ αPD-1	MEK inhibitor/ ICI	Breast cancer cell, Xenograft model	Decreased tumor volume Augmented infiltration of CD4/8+ cells	[30]

Abbreviations: siRNA, Small interfering RNA; shRNA, Short hairpin RNA; PARP, Poly ADP ribose polymerase; EMT, Epithelial–mesenchymal transition; TFBG, Theaflavin-3,3′-digallate; PLK1, Polokinase 1; CBP, CREB binding protein; ICI, Immune checkpoint inhibitor.

## Abbreviations

The following abbreviations are used in this manuscript:

SHCBP1	SHC SH2-domain binding protein 1
mPAL	Murine protein of activated lymphocytes
NSCLC	Non-small cell lung carcinoma
HCC	Hepatocellular carcinoma
CDK	Cyclin-dependent kinase
EMT	Epithelial–mesenchymal transition
PbH1	Parallel beta-helix
PLK1	Polo-like kinase 1
EGF	Epidermal growth factor
TGF-β	Transforming growth factor β
MiRNA	MicroRNA
LncRNA	Long noncoding RNA
CeRNA	Competing endogenous RNA
AURKB	Aurora kinase B
FGF	Fibroblast growth factor
USP49	Ubiquitin-specific peptidase 49
CBP	CREB binding protein
HNSCC	Head and neck squamous cell carcinoma
VEGF	Vascular endothelial growth factor
RTK	Receptor tyrosine kinase
PDAC	Pancreatic ductal adenocarcinoma
EOGT	EGF domain specific O-linked GlcNActransferase
TME	Tumor microenvironment
TAM	Tumor-associated macrophage
TMB	Tumor mutation burden
MSI	Microsatellite instability
CAG	Cancer–associated fibroblast
ICI	Immune checkpoint inhibitor
PPI	Protein–protein interaction
SCLC	Small cell lung cancer
RNAi	RNA interference
SiRNA	Small interfering RNA
ShRNA	Short hairpin RNA
TFBG	Theaflavin-3,3′-digallate
